# Social Determinants of HIV Disparities in the Southern United States and in Counties with Historically Black Colleges and Universities (HBCUs), 2013–2014

**DOI:** 10.1371/journal.pone.0170714

**Published:** 2017-01-20

**Authors:** Madeline Y. Sutton, Simone C. Gray, Kim Elmore, Zaneta Gaul

**Affiliations:** 1 Division of HIV/AIDS Prevention, Centers for Disease Control and Prevention, Atlanta, GA, United States of America; 2 ICF, Atlanta, GA, United States of America; British Columbia Centre for Excellence in HIV/AIDS, CANADA

## Abstract

HIV infection disproportionately affects Blacks in the southern United States (U.S.), a region where legal policies that may unintentionally impede earlier HIV detection and treatment are prevalent. Historically Black Colleges and Universities (HBCUs) have historically facilitated social change in communities of color and have been underexplored as partners for HIV prevention. We describe geographic and social determinants of health (SDH) in the southern U.S. to inform potential HBCU-public health partnerships that might improve HIV health equity. We evaluated the relationship between county-level HIV prevalences (2013), HBCU geographic coordinates, and SDH variables in the southern counties with HBCUs. U.S. Census-derived SDH variables included race/ethnicity (i.e., Black, White, Hispanic), unemployment, female head of household, poverty, percent owner-occupied housing units, urbanicity, and primary care provider rates. Associations were assessed using bivariate and multivariable linear regression. Of 104 HBCUs in the contiguous U.S., 100 (96%) were located in 69 southern counties with average Black populations of 40% and an average HIV prevalence of 615. 5 per 100,000, over two times the national rate (295.1 per 100,000). In bivariable analyses, higher HIV rates in these counties were associated with higher percent Black population, unemployment, female head of household, poverty, fewer owner-occupied housing units, and greater urbanicity (p < 0.05). In multivariable analyses, counties with higher HIV rates had higher percentages of Blacks, greater urbanicity, fewer owner-occupied housing units, and more female heads of households (p < 0.05). The southern U.S. is disproportionately affected by HIV, and many HBCUs are located in affected southern counties. HBCUs may be important public health partners for helping to develop structural interventions that strengthen HIV policies in support of health equity in these southern, mostly urban counties.

## Introduction

The southern United States (U.S.) is disproportionately affected by human immunodeficiency virus (HIV), accounting for 44% of persons living with HIV in 2014 [1]; however, only 38% of the U.S. population resided in the South as of 2015 [2]. Blacks/African Americans (hereafter referred to as Blacks) disproportionately reside in the South and are significantly affected by HIV in the South [3]. In 2011, the HIV diagnosis rate among Blacks in selected southern states (Alabama, Florida, Georgia, Louisiana, Mississippi, North Carolina, South Carolina, Tennessee and Texas) was 73.7 per 100,000, compared with 24.8 per 100,000 among Hispanics/Latinos, and 8.8 per 100,000 among Whites.^3^ Blacks accounted for 54% of new HIV diagnoses in the South in 2014 [1]. In addition, Blacks in the South have later initiation of antiretroviral therapy and greater HIV-related morbidity and mortality compared with non-Hispanic Whites [4]. Recent data also underscore the heightened HIV risk of young black men who have sex with men (BMSM) in the southern U.S., suggesting diagnosed HIV prevalences of ≥ 15–25% in several southern states and urgent action to develop and implement new social and structural interventions strategies [5]. Such strategies are vital to engage BMSM beyond largely suboptimal individual-level HIV risk factor interventions [5, 6].

Many Black communities affected by HIV in the southern U.S. are also disproportionately affected by social and structural determinants of health (SDH) that have historical and political roots of injustice, poverty, racism and unequal opportunities to access education and employment, all of which contribute to HIV-related racial/ethnic disparities and deter from HIV health equity goals, and which have presented challenges for partnerships with institutions that serve historically underrepresented populations [3, 7]. A legal policy which may also impede health equity and healthcare access to facilitate early HIV screening and/or treatment is the lack of Medicaid expansion in the southern United States, especially in the Deep South States of Alabama, Florida, Georgia, Louisiana, Mississippi, North Carolina, South Carolina, Tennessee, and Texas [8, 9]. Addressing these social and structural-level determinants within communities often requires support and buy-in from trusted community partners before meaningful engagement can occur [10].

Historically Black Colleges and Universities (HBCUs), which were established after the Civil War ended in 1865 to provide educational opportunities and combat social injustices for freed slaves and other Blacks/African Americans, are also disproportionately located in the southern United States [11]. The Higher Education Act of 1965 formally defined an HBCU as “any historically Black college or university established prior to 1964, whose principal mission was, and is, the education of Black Americans, and that is accredited by a nationally recognized accrediting agency or association determined by the Secretary [of Education] to be a reliable authority as to the quality of training offered or is, according to such an agency or association, making reasonable progress toward accreditation [12].” HBCUs have traditionally led and/or facilitated social change, movement, and even wellness promotion within communities of color, as trusted partners, while also educating (mostly) Black students in the unique cultural context of support and tradition [13–16]. However, public health partnerships in communities of color hit hardest by HIV have been limited and underutilized, especially with HBCUs.

HBCUs continue to be a valued, trusted resource for educational opportunities for many Blacks [13, 14]. Although some HBCUs have had fiscal challenges and closings and represent only 3% of U.S. colleges and universities, in 2010 HBCUs produced ~37% of Black undergraduates who received bachelor degrees in physical science studies, including public health. Also in 2010, the White House strengthened support of continued HBCU student enrollment by signing an executive order to promote excellence, innovation and sustainability at HBCUs [17], including support for careers in the sciences, public health and public policy [18, 19]. In light of the large proportion of Blacks who receive science-related degrees from HBCUs, engaging HBCU students early in community-level, public health prevention efforts may be another important strategy to consider for long-term domestic health equity goals. Additionally, the historical context and effect of community-level distrust for traditional public health institutions and providers [20, 21] may be lessened by direct engagement and collaborations with local, trusted HBCUs in communities affected by public health disparities.

To date, HBCUs have been underexplored as public health partners for HIV prevention in communities where they are located. Current models to address community-level health disparities include a few early inter-university (between two or more universities) partnerships to inform disparities-focused science and contribute to a more diversified workforce [22, 23], but inadequate support for institutional infrastructure and lack of data showing sustained, scientific progress remain as challenges. In addition, southern HBCUs are noted in only four of these inter-university partnerships to date (total of 18 partnerships were funded), and the federally funded work ranges from public health to clinical science for many diseases, including HIV infection, so the partnerships are not HIV-specific. (Inter-university partnerships were noted at: 1- Shaw University (HBCU) and University of North Carolina at Chapel Hill in North Carolina; 2-Morehouse School of Medicine (HBCU) and Emory University in Georgia; 3-Meharry Medical College (HBCU) and Vanderbilt University in Tennessee; 4-Howard University (HBCU) and Georgetown University in Washington, D.C.) Available county-level social and structural data and geospatial data may provide additional context for defining the inter-related networks of SDH, geography, and HIV rates, and may strengthen support for long-term, inter-university and community-level public health intervention partnership opportunities to reach health equity goals.

To provide data in support of future partnerships, we hypothesized that: 1) HBCUs are located in communities that are disproportionately affected by HIV and SDH, especially in the South, and 2) geographic approaches to reviewing these data will support efforts to strengthen HBCU-public health partnerships to decrease HIV-related racial/ethnic disparities and improve HIV-related health equity. By developing and strengthening national and local public health partnerships with HBCU’s, we can potentially close the gaps on the disproportionate rates of HIV in many disproportionately affected Black communities.

## Methods

### HIV Surveillance Data

County-level estimates of HIV prevalences (Blacks per 100,000 Black population) in 2013 were obtained from the Centers for Disease Control and Prevention’s (CDC’s) National HIV Surveillance System’s (NHSS), National Center for HIV, Viral Hepatitis, Sexually Transmitted Diseases, and Tuberculosis Prevention (NCHHSTP) Atlas. The NCHHSTP Atlas is available at: http://gis.cdc.gov/grasp/nchhstpatlas/main.html?value=atlas. The cumulative surveillance data are restricted to the population aged ≥13 years and reported to CDC through year-end 2013. In the NHSS, rates were calculated for each county using population denominators from the U.S. Census Bureau and data were statistically adjusted for reporting delays [24]. A data suppression rule is applied to county-level HIV surveillance data if the population denominator is less than 100 or total case count less than 5. In accordance with the federal human subjects protection regulations [25] and guidelines for defining public health research [26], NHSS was determined to be a non-research, public health surveillance activity used for disease control program or policy purposes.

**Fig 1:**
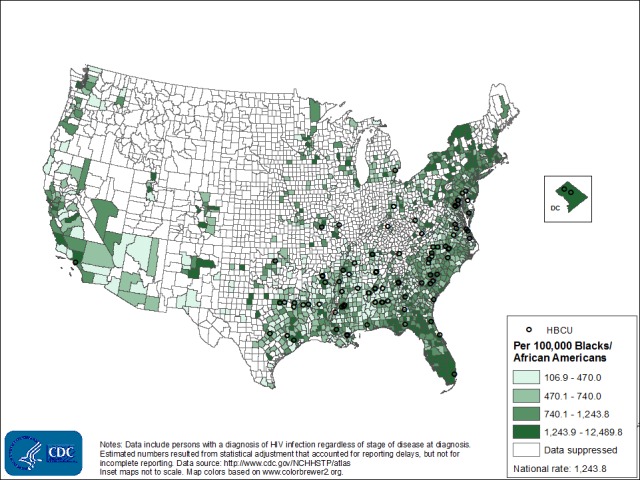
Estimated Rates of Blacks/African Americans Living with Diagnosed HIV Infections, 2013, and Locations of HBCUs, U.S.

The analysis was restricted to only counties in the U.S. South, based on the census-defined “South” region. Census geographic definitions are available at: https://www.census.gov/popest/about/geo/terms.html. The “South” includes Alabama, Arkansas, Delaware, Florida, Georgia, Kentucky, Louisiana, Maryland, Mississippi, North Carolina, Oklahoma, South Carolina, Tennessee, Texas, Virginia, West Virginia, and the District of Columbia. To map the HBCUs, we used ArcGIS 10.3.1 software to geocode the street addresses of the HBCUs (ESRI, Redlands, CA). The distribution of HIV prevalence and locations of HBCUs in the U.S. South were mapped using ArcGIS 10.3.1 as well and are shown in Fig 1.

### US Census Data

We acquired SDH of health variables from the 2010–2014 U.S. American Community Survey (ACS) [27]. The ACS is a national, ongoing survey that uses continuous measurement methods with samples of persons based on census data. ACS data provide vital demographic and social information about people in the U.S. The 5-year estimates from the ACS are "period" estimates that represent data collected over a period of time; using these multiyear estimates increases statistical reliability of the data for less populated areas and small population subgroups.

For this analysis, county-level SDH variables obtained included: race/ethnicity (percentage of non-Hispanic Blacks, non-Hispanic whites, and Hispanics); poverty (percentage of county population below the federal poverty level; the federal poverty level (FPL) is the set minimum amount of gross income that a family needs for food, clothing, transportation, shelter and other necessities. In the U.S., the FPL is an indicator to define “poor” and is determined by the Department of Health and Human Services annually); income (household median income); education (percentage of persons aged ≥ 25 years with less than a high school education); unemployment (percentage of non-institutionalized population unemployed); home ownership (percentage of owner occupied homes); female head of household (percentage of total families with children under 18 and a female head of household); urbanicity (assigned counties a value of 1 if they had at least 50,000 inhabitants to be considered “urban” and 0 otherwise); and primary care providers (primary care physicians and mid-level clinicians per 100,000 population).

### Statistical Methods

We evaluated the relationship between log-transformed county-level HIV prevalences and each independent SDH variable using bivariable linear regression models for all 69 counties in the US South with HBCUs. A multivariable regression model was used to examine joint SDH factors associated with the HIV prevalence outcome. The best subset selection method was used to determine the variables included in the final adjusted model with criteria based on the smallest Akaike’s Information Criteria (AIC) [28]. Adjusted estimates and 95% confidence intervals (CI) were calculated. All analyses were performed using SAS 9.3 (SAS Institute, Cary, NC).

## Results

**Fig 2:**
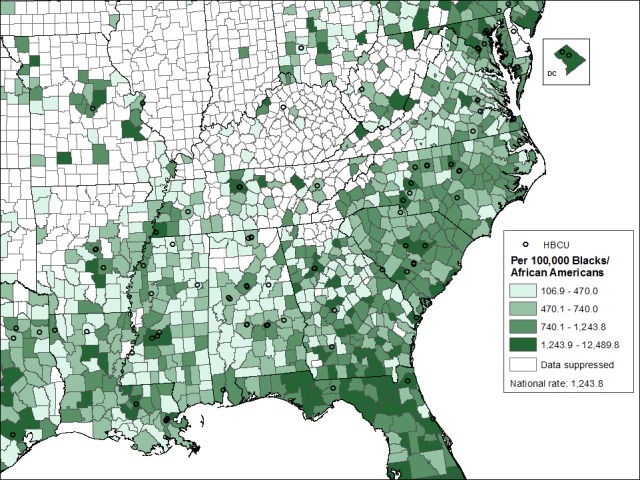
Estimated Rates of Blacks/African Americans Living with Diagnosed HIV Infections, 2013, and Locations of HBCUs, Southern U.S.

Of 104 HBCUs in the contiguous U.S., 100 (96%) were located in 69 southern counties, and those counties have disproportionately higher rates of HIV per 100,000 Blacks (Fig 1). A closer look at the distribution of HIV rates per 100,000 Blacks in the southern counties is shown in Fig 2. Approximately 80% (n = 55) of the southern counties with HBCUs have rates of Blacks living with HIV above the average rates for whites and Hispanics in the south. The average rate for Blacks in the South in 2013 was 831.0 per 100,000 compare to 229.9 per 100,000 for whites and Hispanics races.

Table 1 summarizes key demographic and SDH variables for the 69 counties with HBCUs. During 2010–2014, these counties had an average Black population of 40.2%, median household income of $44,597, unemployment rate of 11.01%, female head of household rate of 36.1%, 20.94% living below the poverty level, a primary care provider rate of 55.6 per 100,000, and an average HIV rate of 615. 5 per 100,000. This HIV rate was more than two times the national HIV prevalence of 295.1 per 100,000 population (2013) [1].

**Table 1:** Demographics of the Southern Counties with Historically Black Colleges and Universities (HBCUs), American Community Survey, United States, 2014 (n = 69 counties).

Variable	Mean	Standard Deviation	Minimum	Maximum
Percent Black	40.20	20.70	5.30	85.90
Percent White	56.20	20	13.7	93.9
Percent Hispanic	8.30	10.80	0.00	65.20
Median Household Income	$44596.97	$9937.10	$23480.00	$73856.00
Unemployment Rate	11.01	3.13	5.90	19.40
Percent Owner-occupied Housing Units	61.73	9.29	41.60	80.70
No high school degree/education	9.96	2.81	4.40	15.90
Percent Uninsured	16.16	3.92	6.40	28.30
Percent Female Head of Household	36.10	6.31	21.80	57.50
Percent below Poverty Level	20.94	6.82	9.70	47.90
Urban, n (%)	15 (21.74)			
Percent Male	48.37	1.37	45.90	54.90
Percent Female	51.62	1.37	45.10	54.10
Primary Care Provider Rates	55.55	26.82	10.10	135.94
Percent Vacant Housing	14.26	5.03	7.40	30.60
Overall rate of HIV(per 100,000 population)	615.45	488.19	56.90	2695.80

In bivariable analyses, higher HIV rates in these counties were associated with higher percent Black population (p < 0.0001), lower percent white population (p < 0.0001), higher unemployment (p = 0.024), fewer owner-occupied housing units (p < 0.0001), higher percent female head of household (p < 0.0001), greater percentages of persons living below poverty (p < 0.0251), and greater urbanicity (p = 0.0005) (Table 2). In multivariable analyses, counties with higher HIV rates had higher percentages of Blacks, greater urbanicity, fewer owner-occupied housing units (all with p < 0.0001), and greater female heads of household (p = 0.026).

**Table 2:** Models of County-level Correlates of Higher HIV Rates, Southern Counties with Historically Black Colleges and Universities (HBCUs), American Community Survey, United States, 2014 (n = 69 counties).

	Bivariable Models		Multivariable Model	
	Outcome: Log (HIV Diagnosis Rates)		Outcome: Log (HIV Diagnosis Rates)	
Predictor	Estimate (95% CI)	p-value^a^	Estimate (95% CI)	p-value
Percent Black	0.02 (0.01, 0.03)	**<0.0001**	0.02 (0.01, 0.02)	<0.0001
Percent White	-0.02 (-0.03, -0.02)	**<0.0001**		
Percent Hispanic	0.01 (-0.01, 0.02)	0.43		
Median Household Income	-0.000 (—)	0.8178		
Unemployment Rate	0.06 (0.01, 0.12)	**0.024**		
Percent Owner-occupied Housing Units	-0.06 (-0.07, -0.05)	**<0.0001**	-0.02 (-0.04, -0.01)	0.004
Education	0.01 (-0.06, 0.07)	0.8202		
Percent Uninsured	0.004(-0.04, 0.05)	0.8705		
Percent Female Head of Household	0.07 (0.05, 0.10)	**<0.0001**	0.01 (0.003, 0.05)	0.026
Percent below Poverty Level	0.03 (0.004, 0.05)	**0.0251**		
Percent Male	-0.10 (-0.23, 0.030)	0.133		
Urban	0.74 (0.33, 1.14)	**0.0005**	0.55 (0.31, 0.79)	<0.0001
Primary Care Provider Rates	0.01 (0.00, 0.02)	**0.0727**		
Percent Vacant Housing	0.02 (-0.02, 0.05)	0.31		

^a^Candidate variables used in the Multivariable model have bolded p-values. CI = confidence interval.

## Discussion

The high rates of HIV diagnoses in the southern U.S. are alarming, especially among Blacks; new, innovative strategies as additional tools to fight the HIV epidemic are warranted. These strategies should include strong, bidirectional and sustainable public health partnerships with HBCUs. Most HBCUs are located in southern counties that are disproportionately affected by HIV and SDH that impede HIV-related health equity; there are likely untapped public health partnership opportunities for community-level HIV awareness, prevention, care, and workforce integration with HBCUs.

Our findings of higher HIV rates in areas with greater urbanicity, fewer owner-occupied units, and more female heads of household suggest that these social and structural factors play a role in the HIV epidemic in these southern counties. These findings are consistent with other analyses which have shown urbanicity and poverty (as associated with less homeownership) to be contributing factors in driving HIV trends in the U.S. [29], especially in the South. In addition, having more female heads of household has been shown to contribute to concurrent sexual partnerships and HIV transmission in some Black communities in the South, due in part to fewer male partners for heterosexual women [30, 31]. To date, community-academic partnerships have been described in southern, rural communities [32] and one Midwestern, urban community [33], but no examples have been published regarding community-academic HIV prevention public health partnerships in the urban South. Based on our findings, addressing HIV in the urban South will require broader interventions that embrace social and structural approaches for solutions and include equitable economic opportunities and gender- and culturally appropriate sexual health messaging. Such approaches are also consistent with the Healthy People 2020 (HP2020) goals’ framework of social justice, which heightens the importance of health equity actions for areas that have been subjected to social and systematic disadvantages, like the southern U.S [34, 35]. For HIV, the overall HP2020 goal is to “prevent HIV infection and its related illness and death [36].”

Our data highlight the importance of increased approaches to engage affected communities in HIV prevention efforts. Innovative solution strategies are warranted, and may include partnerships that embrace and showcase trusted HBCU partners for increased discussions and proposed solutions toward HIV equity. Recent efforts by public health partners, including the Morehouse School of Medicine [37, 38] and the Black AIDS Institute [39] included working with administrators and students at HBCUs and surrounding communities in an effort to increase awareness about the broader HIV epidemics that disproportionately affect some Black communities and increase buy-in for national efforts toward reducing HIV-related health disparities goals. Also, in 2015, the White House hosted a national strategy update meeting at Morehouse School of Medicine, an HBCU medical school, which highlighted that we still have “an HIV epidemic, and that the risk to young Black, gay men remains severe [38].” (https://www.youtube.com/watch?v=AKLD9xaWHwI). Consistent with HIV data showing alarming racial/ethnic disparities for MSM in the South, with young, Black MSM being particularly hard hit [5, 6], our HIV prevention and care efforts with young, Black MSM must be comprehensive, and include broader social and structural-level interventions that include reducing HIV-related stigma and homophobia, including at HBCUs [40–42].

Beyond increasing awareness, efforts are needed to encourage a more diversified workforce through HBCU partnerships to be more actively engaged in public health and policy solutions toward HIV equity, consistent with the National HIV/AIDS Strategy [43, 44]. Although HBCUs continue to be recognized as trusted community leaders and support venues for historically underrepresented students who disproportionately enter the sciences workforce, and some federal public health partnerships have been described to expose historically underrepresented students to public health through extended training opportunities [45, 46], more may be needed to address the heightened HIV burden in many southern HBCU counties [47, 48]. To date, only four HBCUs offer formal master of public health (MPH) training programs: Morehouse School of Medicine in Atlanta, GA; Charles R. Drew University of Medicine and Science in Los Angeles, CA; Meharry Medical College in Nashville, TN; and Florida A&M University (FAMU) offers an online MPH program. HBCUs with and without public health programs can be considered for strengthened partnerships and collaborations to develop more effective HIV prevention interventions for neighboring communities. Some effective programs have engaged student-age peers to convey prevention messages with culturally appropriate content and tone [39, 44]. Interventions such as these could be extended to include HBCU faculty and administrators with culturally based messages and activities based on local epidemiological data and community-engaged strategies. Public health partnerships with HBCUs can be an additional HIV educational, prevention, and care resource for Black communities in these southern, urban counties.

In addition, these county-specific social determinants and HIV prevalence data can be shared with local community leaders and public health practitioners in an effort to have a broader discussion about overlapping health equity needs, how to develop programs and research, and how to implement interventions that address the contributing, underlying social and structural factors. HBCU leaders and community leaders can consider partnerships and develop policies to strengthen HIV prevention and care while developing stronger public health infrastructure. Community-based participatory research with HBCU students, administrators, community members, and local public health leaders can pool resources, collaborate and exchange community-driven ideas for HIV interventions that may resonate best with the affected communities. These discussions can be strengthened by developing maps of HIV prevalences, such as the one developed for this paper; using county-level (or even more local) maps to provide visual context for local HIV epidemics may allow community members to visualize HIV patterns in a way that helps develop and drive their local prevention and care strategies.

## Limitations and Strengths

Our study has limitations. First, the surveillance estimates and SDH variables used in these analyses represent brief snapshots in time and do not fully account for the multilevel, complex associations that exist when considering HIV and SDH associations; no causal relationship can be inferred. Future efforts should measure and include more SDH variables measured over a period of time. Second, not all HIV surveillance data are mapped, often due to missing or incomplete county-level addresses, so there are likely cases excluded and not able to be counted as part of the mapping process. This may result in an under-estimation of HIV cases, which can negatively affect our findings Third, no comparison with non-HBCU counties was conducted in these analyses; comparisons with non-HBCU counties should be considered for future analyses. Fourth, ACS data represent national estimates based on the sampling process through the U.S. Census; improving these estimates requires strengthening efforts to increase response rates for the census surveys. A major strength of this report is that it uses multi-level statistical methods to examine the relationship between SDH and HIV infection in disproportionately affected southern U.S. counties with HBCUs; these methods can inform future studies and social and structural interventions for improved HIV prevention and care in the South.

## Conclusions

The disparities in HIV diagnoses, treatment, and care noted in the South are long-term and rooted in many complex historical, interconnected social and structural determinants of health. This U.S. southern context continues to be challenged with racism, discriminatory policies and practices, disproportionate community levels of sexually transmitted diseases, including HIV, unequal access to education, low health insurance rates, lack of Medicaid expansion opportunities, and high rates of incarceration and unemployment [7, 49]. Of 104 HBCUs in the contiguous US, 100 (96%) were located in 69 southern counties with average Black populations of 40% and an average HIV prevalence of 615.45 per 100,000, over three times the national rate (295.1 per 100,000). Because of their long history as educational and support systems in Black communities, HBCUs may be well-positioned to help navigate and bridge some of the access, public health education, and messaging that will be vital as part of HIV prevention strategies in the South [50, 51]. HBCUs may also be important partners for strengthening HIV policies that favor health equity and for challenging policies that impede progress. Increased public health interventions that engage HBCUs in HIV prevention are warranted, especially in highly affected southern communities. Programs that create federal, local health department, and/or interinstitutional academic partnerships that are funded, sustainable and that strengthen the foundation for primary research and community-engaged partnerships at HBCUs are crucial as additional tools to be explored for HIV awareness, education, prevention, care, and policy development toward improved health equity in the southern U.S.
